# Converging flow and anisotropy cause large-scale folding in Greenland's ice sheet

**DOI:** 10.1038/ncomms11427

**Published:** 2016-04-29

**Authors:** Paul D. Bons, Daniela Jansen, Felicitas Mundel, Catherine C. Bauer, Tobias Binder, Olaf Eisen, Mark W. Jessell, Maria-Gema Llorens, Florian Steinbach, Daniel Steinhage, Ilka Weikusat

**Affiliations:** 1 Mineralogy and Geodynamics, Department of Geosciences, Eberhard Karls University Tübingen, Wilhelmstrasse 56, 72074 Tübingen, Germany; 2 Glaciology Department, Alfred Wegener Institute, Helmholtz Centre for Polar and Marine Research, Am Alten Hafen 26, 27568 Bremerhaven, Germany; 3 Paleoanthropology, Senckenberg Center for Human Evolution and Paleoenvironment, Eberhard Karls University Tübingen, Rümelinstrasse 23, 72070 Tübingen, Germany; 4 Department of Geosciences, University of Bremen, PO Box 330 440, Bremen 28334, Germany; 5 Centre for Exploration Targeting, School of Earth and Environment, The University of Western Australia, 35 Stirling Highway, Crawley, Crawley, Western Australia 6009, Australia

## Abstract

The increasing catalogue of high-quality ice-penetrating radar data provides a unique insight in the internal layering architecture of the Greenland ice sheet. The stratigraphy, an indicator of past deformation, highlights irregularities in ice flow and reveals large perturbations without obvious links to bedrock shape. In this work, to establish a new conceptual model for the formation process, we analysed the radar data at the onset of the Petermann Glacier, North Greenland, and created a three-dimensional model of several distinct stratigraphic layers. We demonstrate that the dominant structures are cylindrical folds sub-parallel to the ice flow. By numerical modelling, we show that these folds can be formed by lateral compression of mechanically anisotropic ice, while a general viscosity contrast between layers would not lead to folding for the same boundary conditions. We conclude that the folds primarily form by converging flow as the mechanically anisotropic ice is channelled towards the glacier.

## Abstract (web summary)

A range of mechanisms has been proposed for large-scale folding in polar ice sheets. Here, using new three-dimensional reconstructions of such folds in the onset region of the Greenland Petermann Glacier, the authors show that these formed due to flow convergence and the high mechanical anisotropy of ice.

## Graphical abstract

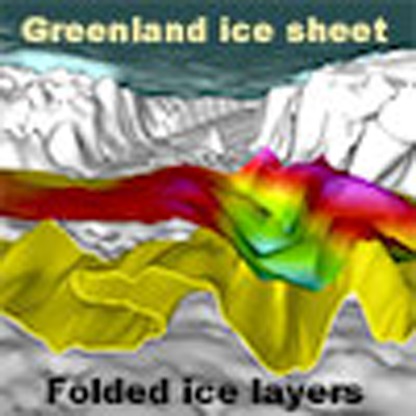

The polar ice sheets of Greenland and Antarctica consist of layer on layer of buried snow, compacted to ice. The ice sheets flow towards their margins under their own weight. Hence, their shape resembles that of a flat dome, with young ice at the top and old ice at the base that can be accessed by ice-core drilling^1^. This has proven an invaluable source for climate data, dating back into the Eemian interglacial period (>115 ka) in Greenland^2^. However, a growing number of radargrams of improved resolution^3^,^4^ have shown that the stratigraphy is disrupted at deeper levels, which was invisible in older radar data and hence termed the ‘echo-free zone'^5^. Folds, from gentle waves to completely overturned layers and sheath folds have been found^1^,^2^,^6^,^7^, as well as truncated layers and discontinuous patchy reflectors^8^. Understanding these structures is relevant as they may indicate disrupted climate record proxies from ice cores^1^. Furthermore, the existence of folds in the ice-sheet stratigraphy highlights that the ice flow from the centres of the ice sheets towards their margins is not as uniform as many standard models indicate^9^.

Under the framework of Operation IceBridge (NASA), dense radar grids (ca. 8-km spacing) have been flown for selected areas^10^, including upstream of the Petermann Glacier in North Greenland, where folds and disturbed basal ice were revealed^6^,^7^,^8^. Here we used structural modelling software, standard in building three-dimensional (3D) models for theoretical and applied problems^11^,^12^, to create 3D models of stratigraphic levels in the lower half of the ice sheet in that area (Fig. 1a). Consistently recognizable radar reflection horizons were traced manually and subsequently combined to form surfaces based on standard interpolation algorithms^13^. In this study, we focus on the area upstream of the Petermann Glacier, where folded layers have been observed^6^,^7^,^8^, and where the spacing of the radargrams and their quality and detail is sufficient to create 3D models of the stratigraphy. Folds can be observed further upstream from our study area and in other areas as well^1^,^7^,^8^, but either the radargram coverage is too sparse or the radar data quality is insufficient to create reliable 3D models.

Here we show that the processes leading to our observed structures are most likely initiated due to the anisotropic nature of ice. To account for the anisotropy, several fully anisotropic flow models have been developed and numerically implemented^14^,^15^,^16^,^17^,^18^,^19^,^20^. However, models considering strain-induced fabric evolution are generally still too computationally expensive to be used on larger scales, and have been mostly applied to small areas or simple geometries of ice divides^14^,^16^,^18^,^19^. Our findings underline the importance of the implementation of the full anisotropy in flow models to predict areas of possible disturbances of stratigraphy.

## Results

### Folds in base of Holocene ice

**Figure 1:**
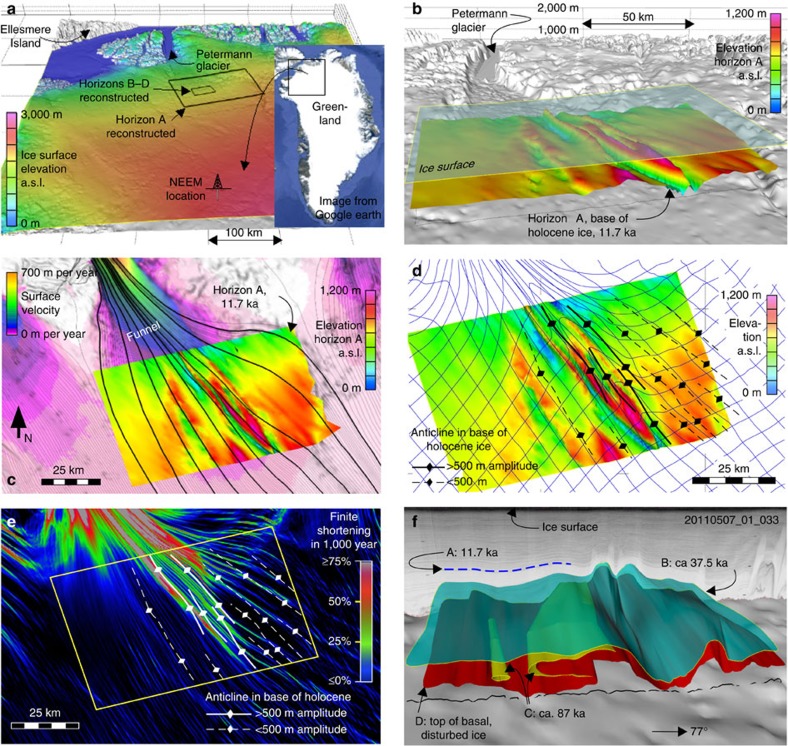
Folds in the lower part of the ice sheet at the onset region of Petermann Glacier. (**a**) Location of the study area in northern Greenland. (**b**) Folds in horizon A at the base of Holocene ice, colour coded for elevation above sea level. (**c**) The same folds (with colour-coded elevation as in **b** shown together with the coloured surface velocity field and flow lines. (**d**) The same folds (colour coded for elevation) now together with a passively deformed grid calculated by integrating the surface velocities^24^,^25^ over a 1,000 years. The original rectangular grid was oriented approximately collinear to the general flow direction. (**e**) Anticlinal hinges (see **d**) shown in relation to the maximum amount of shortening in per cent derived from integration of surface velocities over 1,000 years. **c**–**e** show that folds are parallel to flow and best developed where convergent flow and deformation is the strongest. (**f**) Visualization of folds in horizons B–D in the detail area. **c**–**e** are in 2D map view, while the others are in 3D oblique view with × 10 vertical exaggeration. The fold shape without vertical exaggeration is shown in Supplementary Fig. 3.

A distinct horizon (A) could be traced over the whole study area (Fig. 1a,b; Supplementary Fig. 1; Supplementary Table 1). It represents 11.7 kyr old ice (transition from glacial to Holocene), derived from data presented in Rasmussen and co-authors^21^ at a depth of ∼1,000 m below the ice surface (Supplementary Fig. 2). This horizon shows open, cylindrical folds^22^ with fold axes in the direction of the surface flow (Fig. 1b,c). These structures were described as ‘units of disrupted radiostratigraphy' by Panton and Karlsson^7^. Fold amplitudes reach ∼1 km with wavelengths on the order of 5–10 km. Folding is most intense where the surface streamlines converge at the onset of the highest flow velocities towards Petermann Glacier. To visualize deformation, rather than using existing convergence estimates^23^, we visualize the evolution of an initially rectangular grid (Fig. 1d). Integration of the surface velocity data^24^,^25^ over a thousand years allows us to inspect the approximate finite deformation (Fig. 1d). This shows that the tightest folds are located where the grid is most intensely deformed. Maximum finite shortening over this 1-kyr time interval is in the order of tens of per cent in the highest strain areas, where these folds are located (Fig. 1e).

### Overturned and sheath folds in deepest ice levels

Folding style changes and fold intensity increases with depth. In deeper layers, we identify overturned folds as well as sheath folds. These were traced in a selected area (Fig. 1a). Figure 1f shows the result for three selected horizons: horizon B at an age of ∼37.5 ka (ref. 1) and horizon C at ∼87 ka (ref. 21). Horizon D represents the top of the basal ice in which the stratigraphy is strongly disturbed^6^,^8^. Folds in horizon B mimic those in horizon A above, but are more intense. Folds in horizons C and D show completely overturned limbs and a tubular sheath fold. Fold axes remain parallel to those in the less-folded levels above. The shape of all surfaces A–D in relation to the ice surface and bedrock^26^ is visualized in an animation (Supplementary Movie 1).

## Discussion

Surface velocity data derived from the MEaSUREs data set^24^,^25^ indicate that ice flow is converging and accelerating down flow, towards the glacier trough. The observed fold axes are parallel to the flow direction and perpendicular to the lateral convergence direction, which is needed to push the ice into the bedrock funnel at the entrance of the Petermann Glacier. On the basis of the clear correlation between fold pattern and surface flow field (Fig. 1c–e), we propose that the lateral convergence is the main and first-order cause for the upright folds. This is in contrast to previously proposed mechanisms for the formation of large-scale folds in ice sheets: formation of folds and sheath folds by flow field perturbations, caused by the subglacial environment, such as bedrock topography, slippery patches, melting and refreezing of basal ice and so on^2^,^6^,^8^,^27^, and folding due to large differences in mechanical properties of ice of different ages^1^.

**Figure 2:**
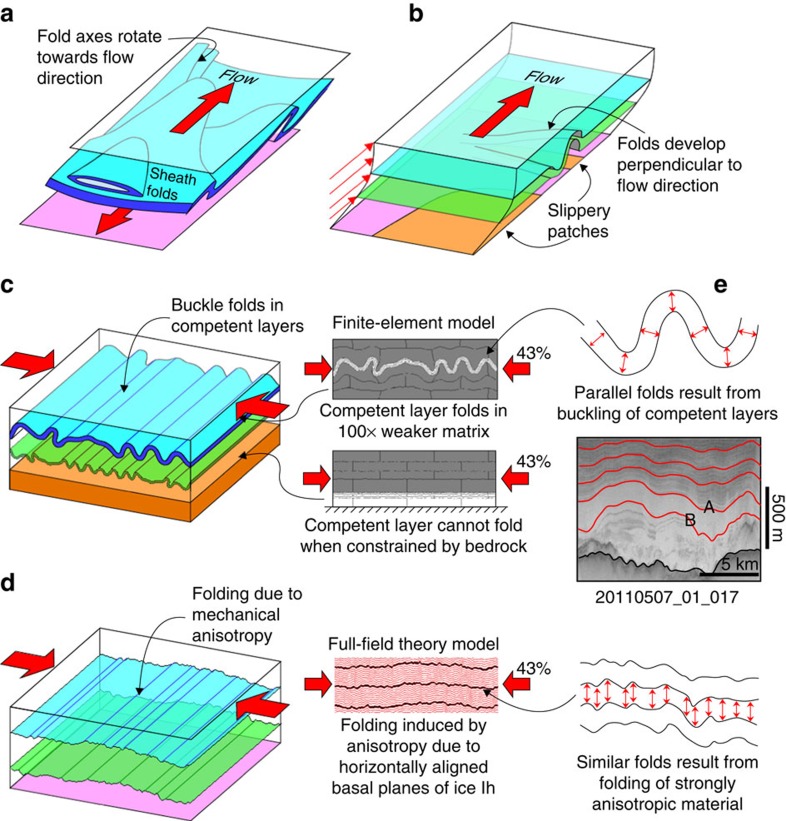
Overview of main relevant fold types and folding mechanisms. (**a**) Sheath folds and overturned folds develop during strong simple shear, as found at the base of ice sheets (after ref. 27). (**b**) Sketch of the expected 3D fold shape when folds develop due to variable basal friction, here illustrated with slippery patches^6^. (**c**) Shape of buckle folds that result from lateral constriction of layers with strong viscosity contrasts, based on our finite-element modelling (Supplementary Fig. 5). (**d**) The simulation of natural fold geometries that result from lateral constriction of a mechanically anisotropic material, based on our full-field theory modelling (Supplementary Fig. 6). (**e**) Comparison of fold shape observed in the radargrams (with horizons A and B labelled) and those resulting from viscosity contrast (**c**) and anisotropy (**d**).

During very strong shearing, all linear structural elements passively rotate towards the shear direction, resulting in fold axes that are parallel to the flow direction and tubular sheath folds^28^,^29^,^30^,^31^ (Fig. 2a). Sheath-fold formation is enhanced if perturbations are amplified due to viscosity contrast between the folding layer and its surroundings^32^ or if the perturbations develop due to a fault, weak layer or shear band^33^. The strong simple shear deformation in ice sheets arises from basal friction that causes a parabolic vertical profile of horizontal flow velocity with simple shear dominating in the lower third of the ice sheet^34^. Sheath folds are thus expected and indeed observed in the lowest levels of the sheet (Fig. 1f), but have so far not been sufficiently investigated.

Slippery patches can develop where the ice sheet is intermittently or not at all frozen to the bedrock, and localize where the pressure melting point is reached or a deformable sediment layer lies between ice and hard bedrock^35^. Wolovick *et al.*^6^ proposed that the variability in bedrock friction caused the overturned folds that are observed upstream of the Petermann Glacier. They based their two-dimensional (2D) model and interpretation on radargrams that are oriented at only a small (25°) angle to the flow direction (Supplementary Fig. 4). In 3D, their model would predict folds with initial fold axes at a large angle (towards 90°) to the flow direction (Fig. 2b). This is inconsistent with the cylindrical folds that we observe to be parallel to the flow direction. The same would apply for folding resulting from basal melting and refreezing^8^. This implies that slippery patches and basal melting/refreezing cannot be the main cause of the upright folds described here.

The incoherence of palaeo-climate data at ∼2,200-m depth in the North Greenland Eemian Ice Drilling core has been attributed to buckle folding that resulted from a large viscosity contrast between glacial and interglacial ice^1^. Buckle folds develop when it is energetically more favourable to accommodate shortening of a competent sheet or layer by bending rather than by homogeneous shortening^27^,^36^,^37^ (Fig. 2c). A viscosity contrast of 50–100 between glacial and interglacial ice was invoked to explain folding in the North Greenland Eemian Ice Drilling core^1^. The initial wavelength (*λ*) of such folds is predicted to be about ten times the thickness of the higher viscous layer^38^,^39^. With *λ*≈5–10 km, the observed folds would need a layer thickness of ∼0.5–1 km, which is roughly one- to two-thirds of the ice sheet thickness in the studied area upstream of the Petermann Glacier. This applies to an isolated layer in an infinite matrix. However, our finite-element modelling shows that the bedrock, which in the model approximately mimics real conditions and acts as a fixed, zero vertical velocity boundary at the base, would hinder the development of folds with the observed amplitudes, even at a viscosity contrast of 100 (Fig. 2c; Supplementary Fig. 5; Methods). We, therefore, regard buckle folding due to a high-viscosity contrast as an unlikely cause for the folding.

To test our hypothesis that convergence flow causes the folds, we consider the highly anisotropic rheology of ice Ih. Slip on its basal plane is ∼60 times easier than on all other slip planes^40^. Polycrystalline ice in ice sheets is known to have a strong lattice preferred orientation locally^41^ as well as along laterally distributed layers^42^, with the tendency for ice basal planes to align perpendicular to the applied finite shortening direction. Under flank flow conditions with shear at the bottom, basal planes are preferably oriented horizontally. Under simple shear along a horizontal plane ice is thus relatively soft (sliding), but strong when shortened in the horizontal direction (causing bending). This rheological anisotropy would cause folding^43^,^44^,^45^ when the ice is shortened parallel to the horizontally aligned basal plane (Fig. 2d). We use numerical full-field theory simulations^45^,^46^,^47^ (see Methods) that take into account the strain-induced crystal-plastic anisotropy of ice Ih to show that even a moderate alignment of basal planes leads to folding (Fig. 2d; Supplementary Fig. 6). A prerequisite for the seeding of folds are small variations in the *c*-axes directions, which are represented in the model as a distribution within a cone of 5° opening angle (see the Methods section). It has to be noted that the simulation of natural fold geometries is not possible in our 2D model. Therefore, some features, such as overturned folds, were not reproduced. The model, however, does show the expected initial fold geometry in a strongly anisotropic material. Fold geometries are expected to be modified in the complex 3D flow field (Fig. 1f) in particular under strong convergence and bedrock-parallel shear.

Folding due to high-viscosity contrasts between layers results in parallel fold*s*^48^, where the strong layers maintain their thickness throughout^27^,^37^ (Fig. 2e). In contrast, our modelling shows that folding due to a rheological anisotropy results in similar folds^48^, where all folded planes have approximately the same shape and fold limbs are, therefore, thinned compared with the hinges. Layer thickness is approximately constant in the direction parallel to the fold axial plane. The folds observed upstream of the Petermann Glacier are close to the similar type (Fig. 2e). Considering the above, we conclude that rheological anisotropy is the favoured cause for folding of ice layers during horizontal shortening as the ice flow converges towards the Petermann Glacier.

**Figure 3:**
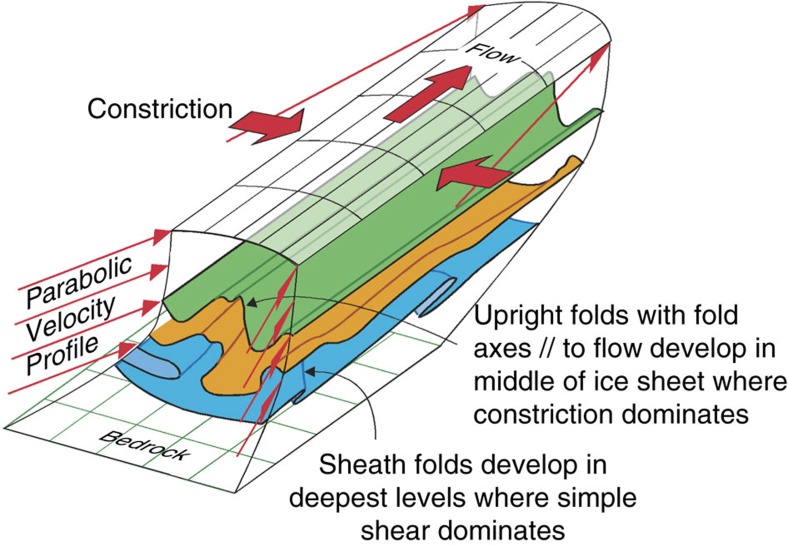
Sketch that summarises the observed folding in relation to constrictional flow towards the Petermann Glacier. Due to bedrock friction, simple shear dominates at the base of the ice sheet, leading to the development of sheath folds. Pure-shear constriction dominates at shallower levels, leading to upright folds with fold axes that are oriented parallel to the flow direction.

In summary, the 3D geometries of folds are visualized in the lower part of the ice sheet upstream of the Petermann Glacier. Sheath folds and overturned folds with fold axes in the flow direction in the very deepest levels (C and D) are interpreted to be the result of the strong bedrock-parallel simple shear component due to basal friction (Figs 2a and 3). Folds at higher levels (A and B) are upright. Their location and orientation relative to the surface velocity field indicate that they result from convergent flow towards the glacier (Fig. 1c–e). Although variable basal friction and basal melting/refreezing may play a secondary a role in fold formation, the dominant mechanism is thus lateral shortening, as the ice flows into the funnel at the entrance of the glacier (Fig. 3). Representations of anisotropy with scalar parameterizations in large-scale ice flow models^15^,^49^ produce variations in effective bulk viscosity, which can result in folding if the viscosity contrast is large enough and would result in parallel-type folds (Fig. 2c). The similar-type folds observed in the inflow region of the Petermann Glacier are best explained as caused by the rheological anisotropy of the ice. To reproduce such folds, the implementation of the full anisotropy is required (Fig. 2d), in addition to minor perturbations in the flow field that trigger fold amplification^38^,^39^, which could be bedrock topography and variations in bedrock sliding^6^,^50^,^51^ or small variations in orientation and intensity of the crystallographic preferred orientation and, hence, in rheological anisotropy of the ice^43^,^45^.

## Methods

### Construction of 3D surfaces

Radargrams from CReSIS were imported as sections into the software 3D-Move (Version 2014), using Polar Stereographic as the coordinate system (Supplementary Fig. 1; Supplementary Table 1). These were combined with the digital elevation models for the bedrock and ice surface^26^. The velocity field and flow lines were derived from refs 24, 25. The selected horizons A–D were traced onto the vertical radargrams that strike 77–257° (Supplementary Fig. 2). We combined the individual traces of the ice horizons using the Discrete Smooth Interpolator method^13^ to create triangulated surfaces in Paradigm GOCAD. A coordinate transform was applied to the traces so that the fold axes were normal to the traces, as this reduces interpolation artefacts. Once the surfaces were created, the inverse transform was applied to restore real-world coordinates. A low-pass filter was then applied to these surfaces to reduce noise. The fly-through movie (Supplementary Movie 1) was created using Avizo 7.1 (FEI Company).

### Finite-element modelling of folding

The 2D finite-element method was used to simulate non-linear viscous deformation in plane strain of a single layer embedded in a softer matrix, as described by Llorens *et al.*^39^ (Supplementary Fig. 5). Both materials are isotropic power-law viscous materials, with a stress exponent of three. The viscosity of the competent layer was set at 100 times that of the weak matrix. Pure-shear velocity boundary conditions were applied to shorten the model by 43%, parallel to the initial layer orientation, in steps of 1% shortening. The model is dimensionless and wavelength of the folds is purely determined by the thickness of the strong layer.

### Full-field theory modelling of folding

Plane strain pure-shear deformation of ice was modelled using a full-field crystal plasticity code^45^,^46^,^47^,^52^ taking into account the viscoplastic anisotropy of ice Ih, with the critical resolved shear stress set 60 times lower for the basal plane than for all other slip planes. The model solves for the stress- and strain-rate field using a fast Fourier transform (FFT) and predicts lattice re-orientations. The model consists of a regular square grid of 256 × 256 Fourier points, each representing a homogeneous volume to which an initial lattice orientation is assigned (Supplementary Fig. 6a). Basal (easy glide) planes were randomly distributed in a cone of 5, 45 and 90° parallel to the horizontal shortening direction (Supplementary Fig. 6b, left column). A total shortening of 43% in steps of 2% was applied to the initial square model, using pure-shear traction and average velocity boundary conditions. As the model material is homogeneous (but anisotropic), deformation is visualized by passively deformed horizontal lines (Supplementary Fig. 6b, right column). Note that, in the absence of any grain scale or compositional or mechanical layering, the model has no absolute length scale.

## Additional information

**How to cite this article:** Bons, P. D. *et al.* Converging flow and anisotropy cause large-scale folding in Greenland's ice sheet. *Nat. Commun.* 7:11427 doi: 10.1038/ncomms11427 (2016).

## Supplementary Material

Supplementary Information Supplementary Figures 1-6, Supplementary Table 1 and Supplementary References.

Supplementary Movie 1 Fly-through movie for the reconstructed folds in the onset region of the Petermann Glacier. The movie shows the geometry of folds in horizons A-D in relation to the ice surface, bedrock and funnel-shaped inlet of the glacier.
