# Supplementary material for: Converging flow and anisotropy cause large-scale folding in Greenland's ice sheet
Source: Nat Commun. 2016 Apr 29;7:11427. doi: 10.1038/ncomms11427 (PMC4855532; doi:10.1038/ncomms11427)
Supplement: Supplementary Information — Supplementary Figures 1-6, Supplementary Table 1 and Supplementary References. [file ncomms11427-s1.pdf]

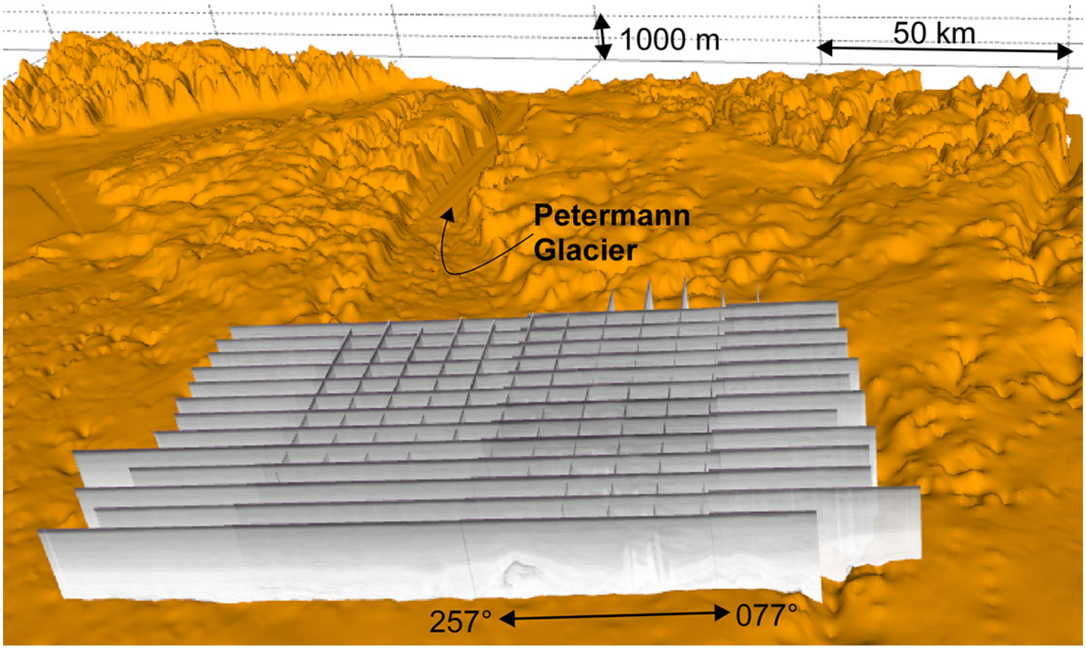

Supplementary Figure 1 | View of the radargrams[1] that were used for drawing traces of the stratigraphic horizons to create surfaces (Supplementary Table 1).

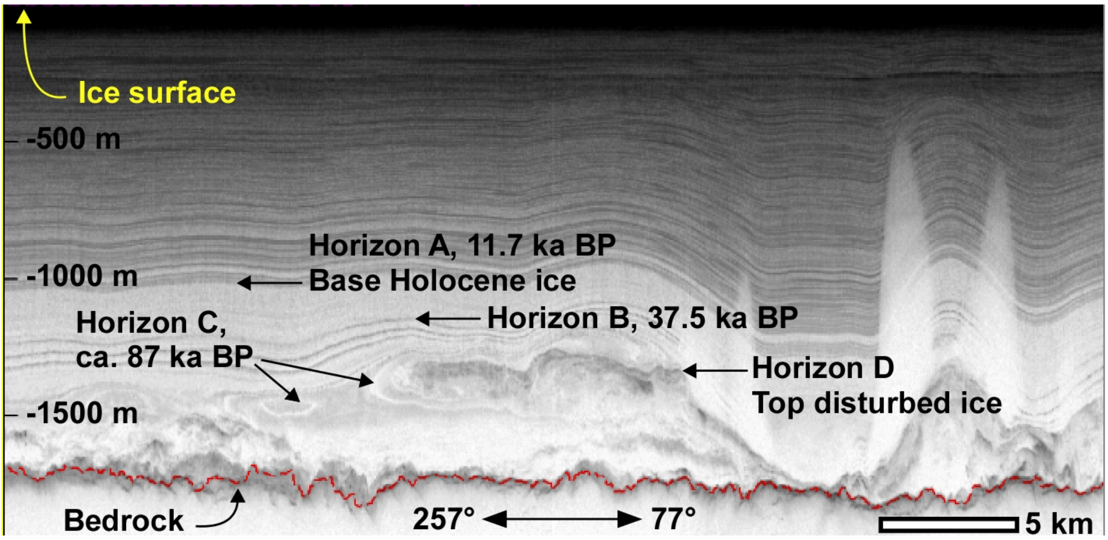

Supplementary Figure 2 | Radargram[1] 20110429_01_010 showing the four selected horizons and their approximate ages[2,3].

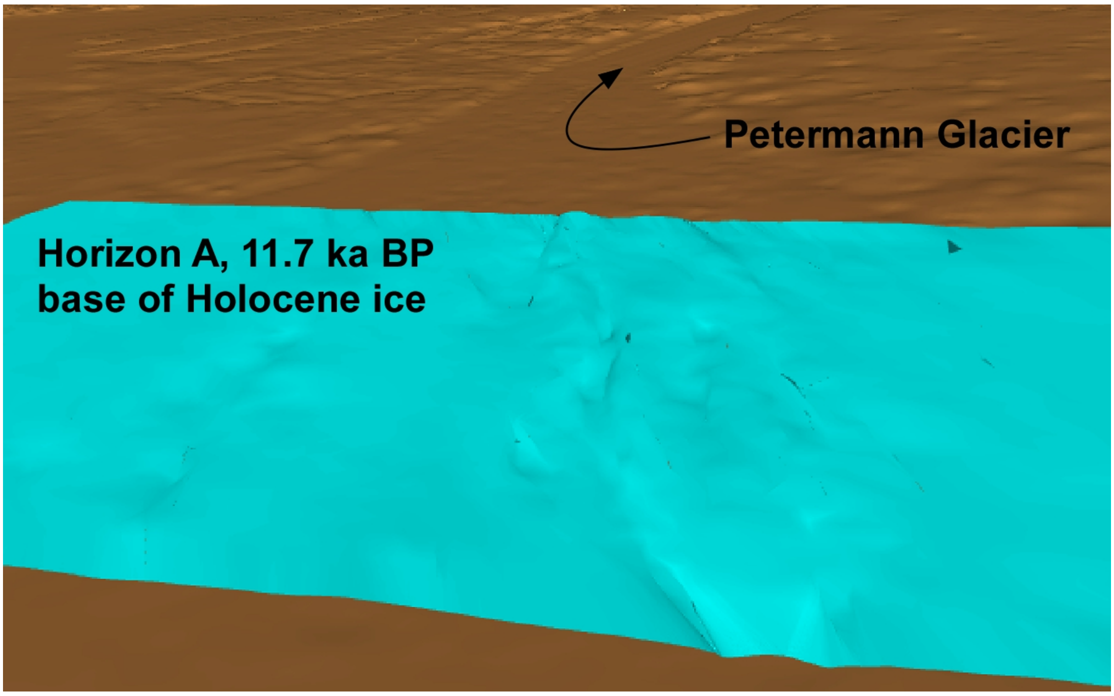

Supplementary Figure 3 | View of the folds without vertical exaggeration. Horizon A, base of Holocene ice, is shown superimposed on the bedrock.

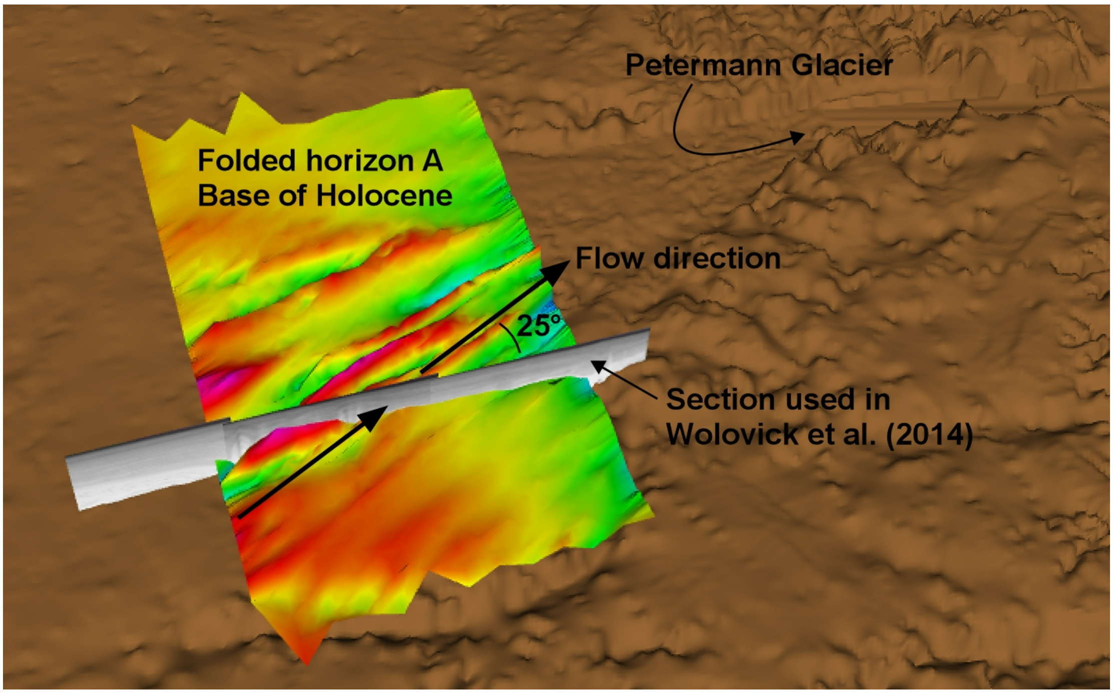

Supplementary Figure 4 | View of radargrams used by Wolovick and co-authors[4] The radargram is shown in combination with the folded surface of horizon A, base of Holocene ice. The radar section used to invoke folding by "slippery patches" is at a small angle to the local flow direction.

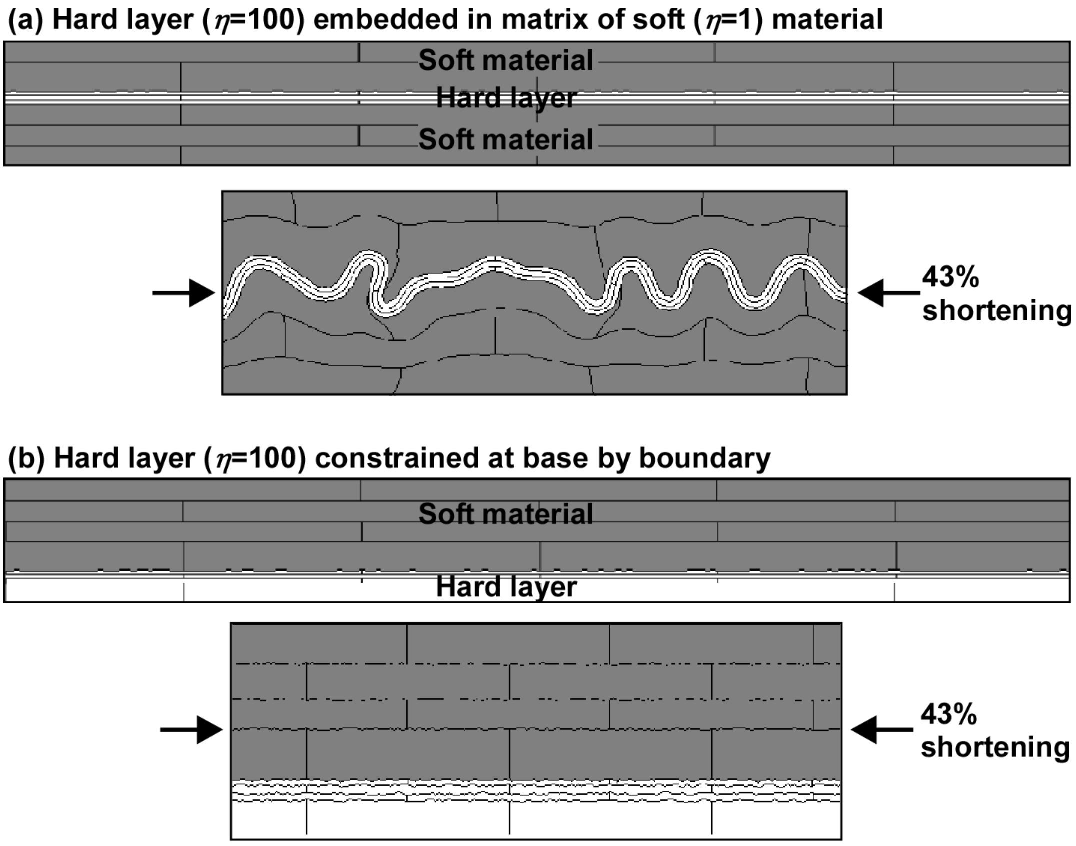

Supplementary Figure 5 | Finite-elements simulation of 43% horizontal shortening of a hard layer and a soft matrix. Both materials are isotropic and have a power-law viscosity with a stress exponent of three. The hard layer (white) has a 100x higher viscosity (η) than the soft material (grey). **a,** The layer is embedded in the matrix and buckle folds develop. **b**, No folds form when the hard layer is constrained at its base.

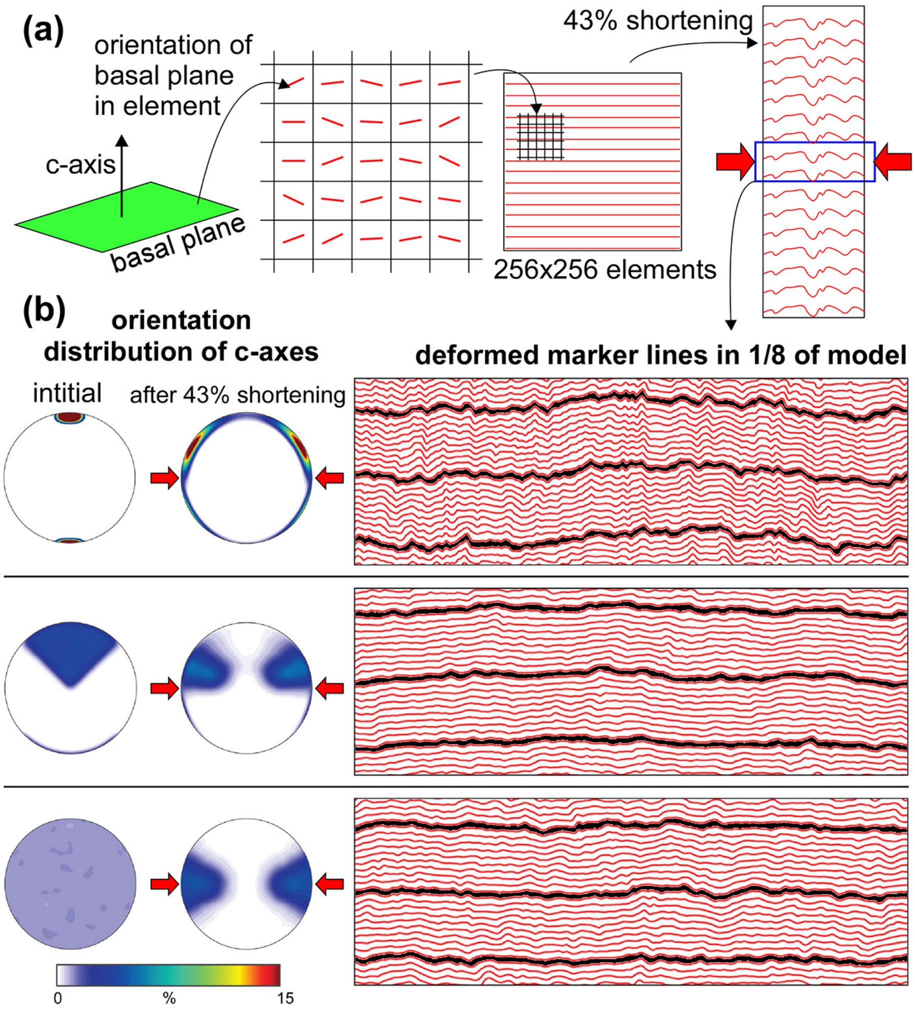

Supplementary Figure 6 | Full-field theory modelling of shortening of anisotropic ice Ih. a, Each dimensionless model consists of 256x256 elements, each with a with an assigned lattice orientation. 43% horizontal pure-shear shortening is applied. **b,** Initial and final distribution of the c-axes (perpendicular to the easy-glide basal plane) is shown on the left. The right column shows that folding of passive marker lines (3 of these highlighted in black) is most pronounced when the basal planes are strongly aligned parallel to the shortening direction (top panel), but even initially random distributions of the basal planes (bottom) leads to minor folding. In three-dimensional flow, with a vertical parabolic velocity field, fold geometry is expected to be more irregular and to include sheath folds, which have not been simulated in this two-dimensional model.

Supplementary Table 1 | Radargrams[1] used in this study. Sections 1 to 13 are oriented 077-257° and sections A to L 164-344°.

Section	Radar data frame no.	Start coordinates	End coordinates
1	20110507_01_011-014	N 79.623°, W 58.224°	N 80.298°, W 51.027°
2	20110507_01_015-018	N 79.560°, W 58.045°	N 80.237°, W 50.870°
3	20110507_01_019-022	N 79.512°, W 57.817°	N 80.182°, W 50.570°
4	20110507_01_023-026	N 79.437°, W 57.625°	N 80.119°, W 50.394°
5	20110507_01_027-030	N 79.387°, W 57.322°	N 80.065°, W 50.100°
6	20110507_01_031-034	N 79.328°, W 57.128°	N 79.998°, W 50.036°
7	20110507_01_035-038	N 79.281°, W 56.803°	N 79.943°, W 49.705°
8	20110507_02_001-004	N 79.212°, W 56.673°	N 79.860°, W 49.823°
9	20110429_01_009-012	N 79.075°, W 57.150°	N 79.826°, W 49.288°
10	20110429_01_013-016	N 79.094°, W 56.247°	N 79.758°, W 49.193°
11	20110429_01_017-020	N 78.991°, W 56.429°	N 79.695°, W 49.018°
12	20110429_01_021-024	N 78.968°, W 55.903°	N 79.668°, W 48.443°
13	20110429_01_025-028	N 78.865°, W 56.082°	N 79.529°, W 49.187°
A	20100324_01_033-034	N 80.256°, W 58.500°	N 79.237°, W 54.780°
B	20100324_01_030-031	N 80.171°, W 57.559°	N 79.305°, W 54.482°
C	20100324_01_023-024	N 80.209°, W 57.114°	N 79.322°, W 54.006°
D	20100324_01_020-021	N 80.418°, W 57.324°	N 79.398°, W 53.715°
E	20100324_01_017-018	N 80.440°, W 56.825°	N 79.484°, W 53.453°
F	20100324_01_014-015	N 80.398°, W 56.053°	N 79.442°, W 52.793°
G	20100324_01_011-012	N 80.524°, W 55.948°	N 79.450°, W 52.288°
H	20110507_02_017-020	N 80.555°, W 55.460°	N 79.142°, W 50.885°
I	20110429_02_006-009	N 80.377°, W 54.199°	N 79.119°, W 50.318°
J	20110429_02_002-005	N 80.417°, W 53.770°	N 79.095°, W 49.744°
K	20110429_01_033-034 & 20110429_02_001	N 80.244°, W 52.561°	N 79.195°, W 49.516°
L	20110429_01_030-032	N 80.287°, W 52.180°	N 79.198°, W 48.996°

Supplementary References

1. Gogineni, P. CReSIS Radar Depth Sounder Data. *Lawrence, Kansas, USA. Digital Media.* http://data.cresis.ku.edu/ (2012).
2. NEEM community members. Eemian interglacial reconstructed from a Greenland folded ice cores. *Nature* **493**, 489–494 (2013).
3. Rasmussen, S.O. *et al.* A first chronology for the North Greenland Eemian Ice Drilling (NEEM) ice core. *Clim. Past* **9**, 2713-2730 (2013).
4. Wolovick, M.J., Creyts, T.T., Buck, W.R. & Bell, R. E. Traveling slippery patches produce thickness-scale folds in ice sheets. *Geophys. Res. Letts.* **41**, 8895-8901 (2014).
